# Altered mitochondrial mass and low mitochondrial membrane potential of immune cells in patients with HBV infection and correlation with liver inflammation

**DOI:** 10.3389/fimmu.2024.1477646

**Published:** 2024-11-22

**Authors:** Liling Ma, Qingzhen Han, Longji Cheng, Huafeng Song, Rui Qiang, Ping Xu, Fei Gao, Li Zhu, Junchi Xu

**Affiliations:** ^1^ Department of Clinical Laboratory, The Affiliated Infectious Diseases Hospital of Soochow University, The Fifth People's Hospital of Suzhou, Suzhou, China; ^2^ Center of Clinical Laboratory and Translational Medicine, The Fourth Affiliated Hospital of Soochow University, Suzhou Dushu Lake Hospital, Suzhou, China; ^3^ Department of Clinical Laboratory, The Affiliated Suzhou Hospital of Nanjing Medical University, Suzhou Municipal Hospital, Suzhou, China

**Keywords:** Mitochondrial mass, low mitochondrial membrane potential, liver inflammation, HBV, T cells

## Abstract

**Introduction:**

Mitochondrial membrane potential (MMP) and mitochondrial mass (MM) affect mitochondrial function and lymphocyte activation, but few studies on HBV infection exist. This study aimed to investigate the regulatory mechanism of mitochondrial dysfunction during HBV infection and its clinical significance by analyzing the alterations of MM and MMP^low^ in peripheral blood immune cells.

**Methods:**

The study enrolled 90 participants, including healthy volunteers(HC) and patients with HBV infection, HBV patients were divided into chronic hepatitis B patients (CHB) and liver cirrhosis (LC) according to the study, and CHB was also divided into an inflammation group and a non-inflammation group. Flow cytometry was used to analyze the changes of MM and MMP^low^ in peripheral blood immune cells. These analyses were correlated with the presence of CHB and LC and indexes related to liver inflammation.

**Results:**

The study revealed significant variations in the percentage of MMP^low^ and MM of CD8^+^T cells associated with the progression of the disease. The MMP^low^ percentage of CD8^+^T cells in the LC group exhibited a notable decrease compared to the HC group and CHB groups. Moreover, MMP^low^ of CD8^+^T cells demonstrated potential in distinguishing CHB and LC (AUC=0.7341, P=0.0032). Furthermore, in exploring the link between mitochondrial function of immune cells and liver inflammation, the study found a negative correlation between the MMP^low^ ratio of CD4^+^T and CD8^+^T cells and AST (p=0.0039 and P=0.0070, r=-0.4405 and r=-0.4146), while the MM of CD8^+^T cells displayed a positive correlation with AST (p=0.0013, r=0.4865). In CHB patients with normal ALT but liver inflammation detected on B-scan ultrasonography, a significant decrease was observed in the MMP^low^ percentage of CD8^+^T (66.13 ± 14.27), CD56^+^NK(57.77 ± 17.40) and CD4^-^CD8^-^T (61.98 ± 15.98) cells. Furthermore, it was also found that the percentage of MMP^low^ in CD4^-^CD8^-^T cells could serve as an indicator for early liver inflammation and injury (AUC=0.8408, P=0.0052).

**Discussion:**

In this study, we conducted a systematic analysis of the percentage of lymphocyte MMP^low^ and MM in various stages of HBV infection. Our findings revealed a correlation between MMP^low^ and MM and early liver inflammation, as well as the progression of the infection. This study marked the first demonstration of the clinical diagnostic value of MMP^low^ and MM in HBV infection. Furthermore, this was the first study to discuss the mitochondria of lymphocytes and liver inflammation in HBV infection. It enhanced the understanding of the role of T cells in liver inflammation, and elucidated potential markers for the early detection of liver injury and clinical cirrhosis.

## 1 Introduction

Mitochondria, an organelle ubiquitously present in most cells, comprises a bilayer membrane structure and matrix component. Mitochondria plays a pivotal role in cellular energy production, metabolism, and signal transmission (1). Any alteration in its structural and functional integrity may lead to mitochondrial dysfunction, characterized by adenosine triphosphate (ATP) depletion, overproduction of reactive oxygen species (ROS), reduction in mitochondrial membrane potential (MMP), and damage to mitochondrial deoxyribonucleic acid (mtDNA). These changes can further perpetuate cell apoptosis and inflammatory response (2). Recent findings suggest that mitochondrial dysfunction arising from mitochondrial injury can trigger a cascade of cell injuries, including ATP depletion, ROS upsurge, inflammatory response, and apoptosis, and is instrumental in the onset and progression of various hepatic diseases such as hepatitis C (3), chronic alcoholic liver disease (4), non-alcoholic fatty liver disease (NAFLD) (5) and drug-induced liver injury (DILI) (6).

Studies have indicated that hepatitis B virus (HBV) infection may result in elevated levels of calcium ions, overproduction of ROS, and reduced ATP synthesis within the mitochondria. Additionally, HBV infection has been linked to the overactivation of the mitochondrial permeability transition pore (MPTP), leading to mitochondrial swelling and cell death (7). Yoo et al. (8) observed a correlation between high expression of MARCH5E3, a mitochondrial ubiquitin ligase, and increased survival rates among patients with hepatocellular carcinoma (HCC). This association is attributed to MARCH5E3’s interaction with the hepatitis B viral x (HBx) protein, which accumulates in the mitochondria and triggers its degradation, thereby inhibiting HBx-induced ROS overproduction, mitochondrial autophagy, and cyclooxygenase-2 expression (8, 9). The above studies confirmed that mitochondria played an important role in HBV infection. Other studies also found circulating lymphocyte activation was associated with HBV infection. These suggested the lymphocyte mitochondria may be abnormal during HBV infection.

Hepatitis B virus (HBV) does not directly inflict damage upon liver cells but does so through the modulation of the immune system (10). T cell activation, proliferation, and differentiation are fundamentally driven by shifting in cellular metabolism, with mitochondria playing a central role (11). Mitochondrial dysfunction, as seen in COX-deficient T cells, can severely impair these metabolic pathways, affect T cell responses, and lead to immunodeficiency due to compromised metabolic functionality (12). Li L et al. (13) found downregulation of the JAK1-STAT3 pathway and depolarization of mitochondria emerged as crucial factors contributing to T cell anergy. Research has demonstrated that the hepatitis B virus can integrate its genetic material into mitochondrial DNA (mtDNA), thereby impacting mitochondrial function (14). Zhou et al. identified mitochondrial dysfunction in the immune cells of individuals with chronic HBV infection, establishing a notable association between mitochondrial function in immune cells and HBV viral load, HBeAg, and HBsAg (15). Presently, limited research exists regarding the connection between mitochondria and immune cells in the context of HBV infection. The impact of HBV infection on T cell mitochondrial function, and T cell exhaustion and differentiation require further investigation. In this study, two novel mitochondrial energy metabolism indices, MM (16) and MMP^low17^ were employed to comprehensively analyze the changes in mitochondrial metabolism of immune cells at various stages of HBV infection. The goal was to explore the correlation between these changes and hepatic inflammation and their potential clinical diagnostic significance. The findings from this research were anticipated to shed light on the immunological mechanisms underlying liver injury resulting from HBV infection, elucidate the variations in immune cell mitochondrial energy metabolism across different stages of HBV infection, introduce novel diagnostic markers for early liver inflammation, and identify new therapeutic targets for HBV-induced liver inflammation.

## 2 Materials and methods

### 2.1 Study population

During December 2023 and January 2024, 90 adults were enrolled in the study from three medical institutions: the First Affiliated Hospital of Suzhou University, the Fourth Affiliated Hospital of Suzhou University, and the Fifth Affiliated Hospital of Suzhou University. Among the participants, 29 were healthy volunteers, and 61 were HBV-infected people. Hepatitis B surface antigen (HBsAg) and hepatitis B core antibody (HBcAb) were positive in all HBV-infected patients and negative in healthy volunteers. In the HBV-infected patients, 27 were HBeAg positive and 34 were negative. According to the Chinese guidelines for the diagnosis and treatment of liver cirrhosis, HBV-infected patients were grouped into chronic hepatitis B patients (n=41) and cirrhosis patients (n=20) based on the clinical symptoms, liver imaging changes, liver function, and liver histology. The exclusion criteria were as follows: i) patients with Immunosuppressive diseases, autoimmune diseases, HDV infection, HCV infection, and other immune-related diseases (malignant tumors, previous transplants, chronic renal failure, and HIV infection); and ii) patients receiving treatment with immunomodulators within a three-month timeframe to avoid confounding immune alterations. The research was carried out in compliance with the ethical standards outlined in 1975 Declaration of Helsinki, and all participants provided their informed consent.

### 2.2 Lymphocyte count and mitochondrial indicator measurements by flow cytometry

Mitochondrial probe (MitoDye) is a cationic dye soluble in fats and possesses both hydrophobic and charged properties. These characteristics allow it to permeate the cell membrane and enter the cell. The dye’s methylene chloride, which reacts with sulfhydryl groups, enables stable binding to proteins in the inner mitochondrial membrane. Accumulation in the mitochondria occurs due to the mitochondrial membrane potential. Thus, the probe can identify and visualize mitochondria within cells and indicate the potential across the mitochondrial membrane. When the mitochondrial membrane potential is high, JC-1 aggregates in the matrix of mitochondria(J-aggregates), which can produce red fluorescence. When the mitochondrial membrane potential is low, JC-1 can not accumulate in the mitochondrial matrix, and JC-1 is monomer and can produce green fluorescence.

2 ml of peripheral blood was collected in tubes coated with EDTA-K2 anticoagulant and detected within 48h by flow cytometry. The detection parameters included percentage, absolute cell count, MM, and MMP^low^ of T cells (CD3^+^), T helper cell subsets (Th, CD3^+^CD4^+^CD8^−^), and cytotoxic T cells (Tc, CD3^+^CD4^-^CD8^+^). Monoclonal antibodies including CD8-FITC (clone: SK1), CD19-FITC (clone: HIB19), CD3-PE (clone: SK7), CD56-PE (clone: HCD56), CD45-PerCP-Cy5.5 (clone: H130), and CD4-PE-Cyanine7 (clone: SK3), along with the mitochondrial detection reagent MitoDye (structural formula C34H36Cl2N2), were obtained from UB Biotechnology Co. LTD (Zhejiang, China).

Of fresh heparinized whole blood, 100 μL was incubated with 20 μL of pre-mixed antibodies for 15 min, then lysed with 2 mL of FACSTM lysing solution (BD Biosciences, San Jose, CA, USA) for 15 min. The sample was centrifuged at 300g for 5 min, the supernatant was removed, and the pellet was resuspended in 100 μL of PBS. Fixation was achieved with 1 μL of MitoDye, followed by incubation at 37°C for 30 minutes. Following sample preparation, labeled immune cells were quantified using flow cytometry (NovoCyte D206) and analyzed with the Human Lymphocyte Mitochondrial Function Analysis System (UB Biotechnology Co. LTD, Zhejiang, China).

At least 20,000 lymphocytes were collected from each sample. The percentage was determined by assessing the ratio of T cells/T subsets to the total number of lymphocytes. The median mitochondrial fluorescence index (MFI) was used to calculate mitochondrial mass, and it was detected by the APC channel of flow cytometry. The percentage of MMP^low^ was analyzed using the mitochondrial-specific marker MitoDye. A Mito/Conte histogram was generated to differentiate between the mitochondrial low and high groups, and the percentage of cells in the low-population group (ratio value) was determined (**Supplementary Figure 1**).

### 2.3 Detection of HBsAg, HBeAg, ALT, AST and γGT

Serological markers of HBV and liver function indicators in serum were measured simultaneously with flow cytometry, including HBsAg, HBeAg, ALT, AST, and GGT. Serum HBsAg and HBeAg were measured using a commercial chemiluminescence kit (Abbott, USA) with an LOD of 0.05 IU/mL HbsAg, positive HBeAg as>1.00 S/CO. Serum alanine aminotransferase (ALT), aspartate aminotransferase (AST), and γ-glutamate transpeptidase (γGT) were determined by Hitachi 7600 biochemical analyzer.

### 2.4 Liver B-scan ultrasonography inspection

CHB patients with normal liver injury indexes (ALT<45 U/L) were divided into an inflammation group(n=14) and a non-inflammation group(n=15) based on the presence of inflammatory changes such as dotted echoic thickening, enhancement, density variations, and uneven distribution in the liver. This categorization was performed by three radiologists.

### 2.5 Data analysis

The statistical analyses were performed using GraphPad Prism 9.0.0 (GraphPad, San Diego, CA, USA) and SPSS 26.0 software. Numerical variable data were expressed as mean ± standard deviation (x̅ ± SD). In cases where the numerical variable data of two groups exhibited normal distribution and homogeneous variance, a t-test was employed; otherwise, the Mann-Whitney U-test was utilized. For numerical variable data involving three groups with normal distribution and homogeneous variance, a One-way ANOVA analysis was performed. If these conditions were not met, the Kruskal-Wallis H test was applied. The receiver operator characteristic curve (ROC) was calculated to evaluate the clinical diagnostic efficacy of immune cell mitochondrial mass (MM) and low mitochondrial membrane potential (MMP^low^) in the context of hepatic injury and progression of HBV. The X-axis of the ROC curve represents 1-specificity (false positive rate, FPR), while the Y-axis represents sensitivity (true positive rate, TPR), AUC: the area under ROC curve. All p values were two-tailed, and values <0.05 were considered statistically significant.

## 3 Results

### 3.1 Analysis of basic clinical data of the study subjects

As shown in **Table 1**, the study enrolled 29 healthy individuals (HC group) comprising 18 males and 11 females with a mean age of 35.45 ± 11.00 years; The CHB group consisted of 41 cases, including 26 males and 15 females, with a mean age of 44.59 ± 12.20 years; Additionally, the LC group comprised 20 cases, with 15 males and 5 females and a mean age of 47.85 ± 11.49 years. Within the CHB group, 40 patients underwent B-scan ultrasonography, revealing liver inflammation in 20 patients. The ALT, AST, and γGT values in the CHB group were 81.34 ± 144.69, 124.94 ± 252.21 and 78.20 ± 162.78, respectively. Furthermore, the ALT and AST values were significantly higher in the CHB group compared to the LC group (58.65 ± 72.53 and 46.85 ± 40.12), with statistically significant differences. Further analysis revealed that the absolute lymphocyte counts s in both the CHB and LC groups were significantly lower than those in HC group, demonstrating statistical differences(**Supplementary Figure 2**).

**Table 1:** Analysis of basic clinical data of the study cohort.

	HC group (29)	CHB group (41)	LC group (20)	p value
Ratio of male	18/29	26/41	15/20	0.902
**Lymphocyte count (10^6/uL)**	**1970.10 ± 476.72**	**1375.78 ± 621.44**	**1038.35 ± 615.66**	**<0.0001**
monocyte count (10^6/uL)	250.10 ± 159.37	176.84 ± 161.35	162.09 ± 231.33	0.1515
Granulocytes count (10^6/uL)	1884.72 ± 1146.34	1596.36 ± 1604.21	1141.25 ± 1230.48	0.1905
CD3^+^CD4^+^T cells count (10^6/uL)	574.86 ± 348.18	463.76 ± 235.11	358.28 ± 212.02	0.0258
**CD3^+^CD8^+^T cells count (10^6/uL)**	**434.74 ± 300.33**	**337.06 ± 174.47**	**216.81 ± 154.76**	**0.0042**
CD3^-^CD19^+^B cells count (10^6/uL)	221.20 ± 132.83	153.82 ± 104.03	215.99 ± 196.82	0.0884
**CD3^-^CD56^+^NK cells count (10^6/uL)**	**541.27 ± 314.75**	**343.81 ± 205.13**	**152.18 ± 96.83**	**<0.0001**
MMP^low^ of lymphocyte (%)	60.94 ± 23.83	65.28 ± 18.13	61.90 ± 16.11	0.6332
MMP^low^ of monocyte (%)	28.62 ± 32.27	14.00 ± 24.22	12.33 ± 24.60	0.0504
**MMP^low^ of Granulocytes (%)**	**44.53 ± 27.80**	**32.80 ± 26.48**	**24.83 ± 24.37**	**0.0347**
**MM of lymphocyte**	**1.025 ± 0.4808**	**0.7851 ± 0.3779**	**0.7580 ± 0.3220**	**0.0165**
MM of monocyte	2.507 ± 1.045	2.400 ± 0.8692	2.179 ± 1.2010	0.5319
MM of Granulocytes	1.741 ± 0.7752	1.455 ± 0.7506	1.549 ± 0.6913	0.2899

The bold values means statistical difference.

### 3.2 Alteration in peripheral blood lymphocyte subsets and mitochondrial activity during different stages of HBV infection

**Figure 1:**
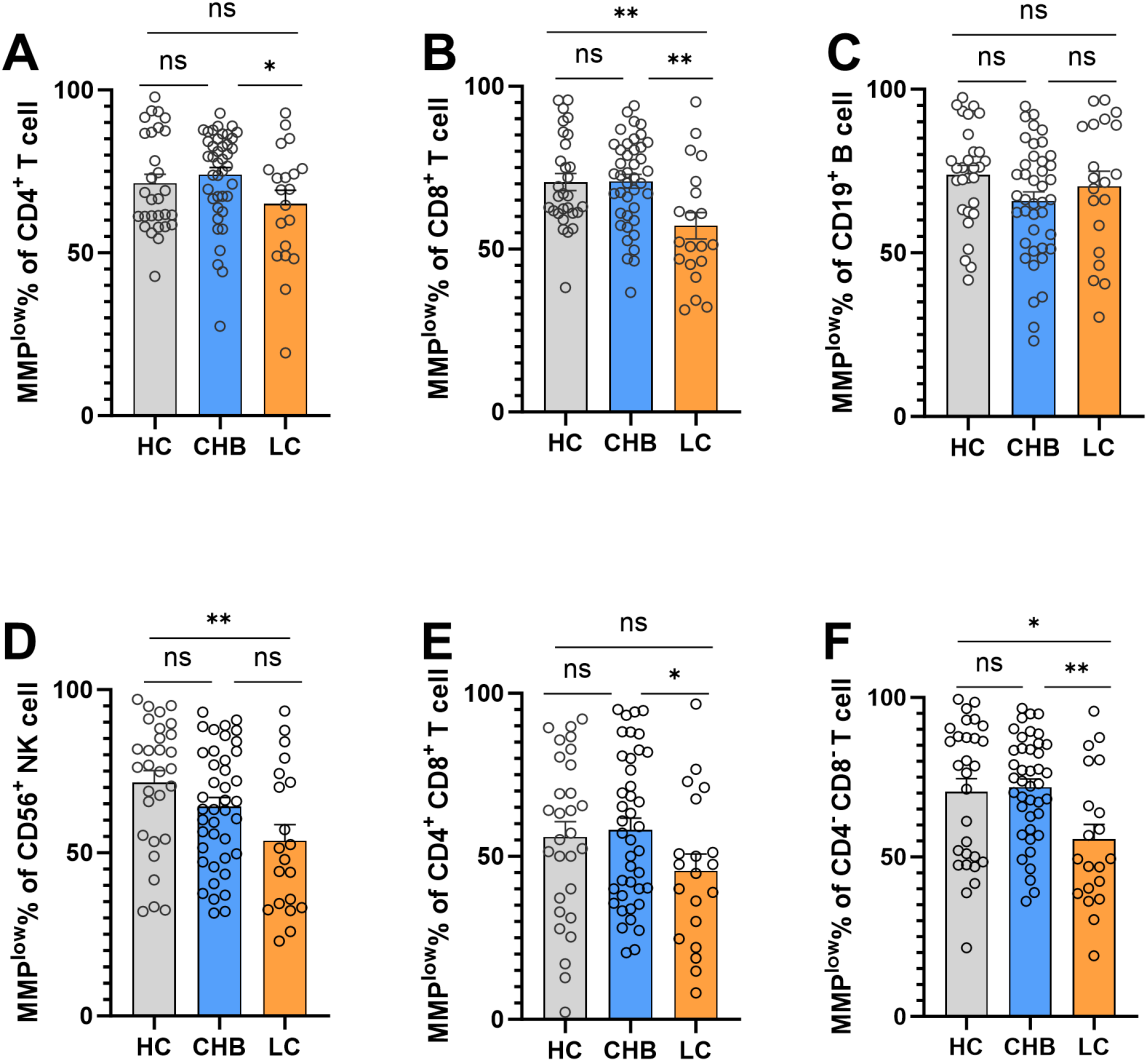
Frequencies of MMP^low^ in the HC group, CHB group and LC group. **(A)** Statistical analysis of the frequencies of MMP^low^ of CD4^+^T cell in the HC group, CHB group and LC group; **(B)** Statistical analysis of the frequencies of MMP^low^ of CD8^+^T cell in the HC group, CHB group and LC group. **(C)** Statistical analysis of the frequencies of MMP^low^ of CD19^+^B cell in the HC group, CHB group and LC group; **(D)** Statistical analysis of the frequencies of MMP^low^ of CD56^+^NK cell in the HC group, CHB group and LC group. **(E)** Statistical analysis of the frequencies of MMP^low^ of CD4^+^CD8^+^T cell in the HC group, CHB group and LC group; **(F)** Statistical analysis of the frequencies of MMP^low^ of CD4^-^CD8^-^T cell in the HC group, CHB group and LC group. *:p<0.05,**:p<0.01 and ns:p>0.05.

Flow cytometry analysis of MMP^low^ of peripheral blood lymphocyte subsets revealed notable findings. The MMP^low^ percentage of CD8^+^T cells in the LC group (57.19 ± 17.99) was significantly lower than that of the HC group (70.58 ± 14.23) and CHB(70.81 ± 13.62) group, and has statistical difference (**Figure 1B**). Furthermore, the percentage of MMP^low^ of CD56^+^NK cells in the LC group (53.72 ± 22.09) was notably lower compared to the HC group (71.52 ± 19.78) (**Figure 1D**). However, no statistical difference was observed in CD4^+^T cells, CD4^+^CD8^+^T cells, CD19^+^B cells and CD4^-^CD8^-^T cells (**Figures 1A, C, E, F**).

In addition, analysis of MM revealed a significantly lower MM of CD4^+^CD8^+^T cells in the chronic hepatitis B group (0.8641 ± 0.3966) compared to the normal healthy control group (1.173 ± 0.5620), with a statistically significant difference (p=0.0088, **Supplementary Figure 3E**). No statistical differences were observed among the three groups in MM of CD4^+^T cells, CD8^+^T cells, CD19^+^B cells, CD56^+^NK cells and CD4^-^CD8^-^T cells (**Supplementary Figure 3**).

The activation of antigen-specific lymphocytes is an immune mechanism in the liver that leads to fibrosis. As HBV disease progresses, immune cells are persistently activated, which is reflected by a continual increase in mitochondrial membrane potential in CD8 and NK cells, known for their high sensitivity to viruses, so the MMP^low^ ratio of CD8 and NK cells decreased in the LC group in our study.

### 3.3 The diagnostic value of MMP^low^ and MM of lymphocyte subsets in delineating various stages of HBV infection

**Figure 2:**
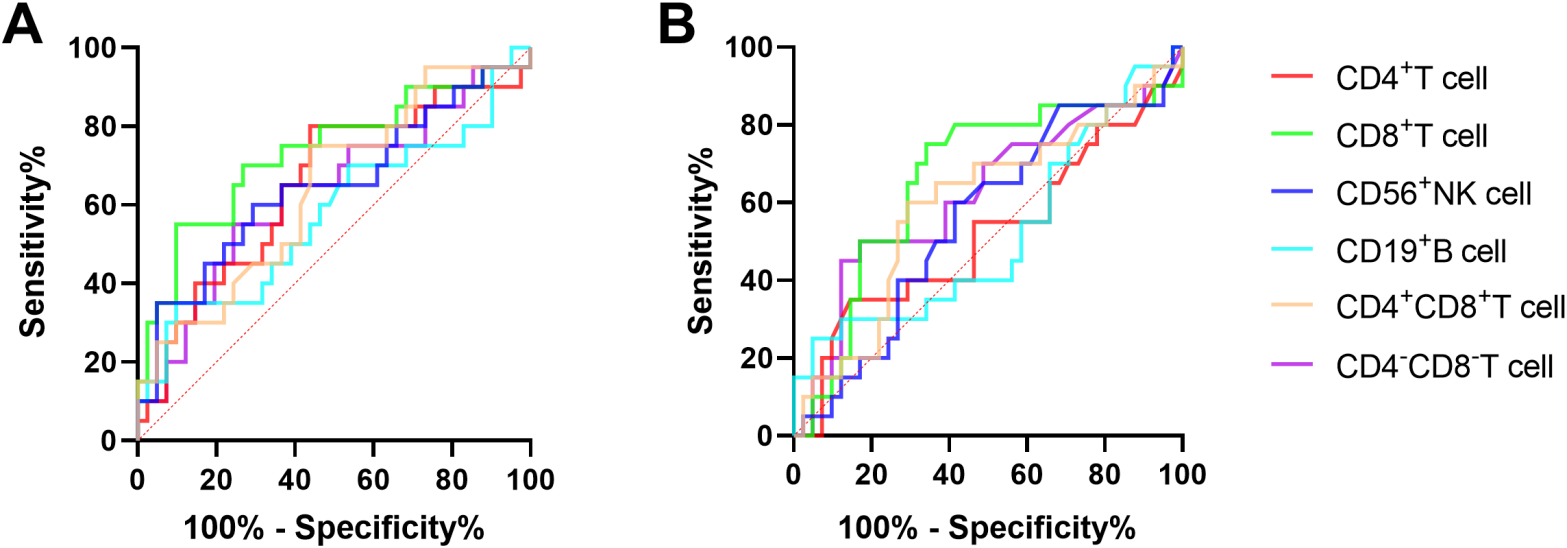
**(A)** The ROC curve of MMP^low^ and MM of immune cells for the difference between CHB and LC patients. **(B)** The ROC curve of immune cells MM for the difference between CHB and LC patients.

The ROC curve analysis was employed to evaluate the discriminatory potential of MMP^low^ and MM in distinguishing patients with chronic hepatitis B from those with cirrhosis. The results indicated that MMP^low^ and MM of CD8^+^T cells exhibited superior discriminatory abilities, with respective AUC values of 0.7341 and 0.6616 and corresponding p-values of 0.0032 and 0.0418 (**Figure 2**; **Table 2**).

**Table 2:** MMP^low^ and MM of lymphocyte subsets distinguished the correlation indexes of ROC curves between CHB and LC patients.

	AUC	95%*CI*	p value
CD4^+^T cell MMP^low^	0.6512	0.4983 to 0.8042	0.0568
**CD8^+^T cell MMP^low^**	**0.7341**	**0.5875 to 0.8808**	**0.0032**
CD19^+^B cell MMP^low^	0.5750	0.4107 to 0.7393	0.3447
CD56^+^NK cell MMP^low^	0.6476	0.4893 to 0.8058	0.0630
CD4^+^CD8^+^T cell MMP^low^	0.6409	0.4917 to 0.7900	0.0760
CD4^-^CD8^-^T cell MMP^low^	0.6329	0.4774 to 0.7884	0.0940
CD4^+^T cell MM	0.5177	0.3515 to 0.6839	0.8237
**CD8^+^T cell MM**	**0.6616**	**0.5056 to 0.8176**	**0.0418**
CD19^+^B cell MM	0.5183	0.3542 to 0.6824	0.8177
CD56^+^NK cell MM	0.5567	0.4019 to 0.7115	0.4750
CD4^+^CD8^+^T cell MM	0.5988	0.4405 to 0.7571	0.2133
CD4^-^CD8^-^T cell MM	0.6207	0.4607 to 0.7808	0.1283

The bold values means statistical difference.

### 3.4 The correlation of mitochondrial activity of lymphocyte subsets with liver injury

**Figure 3:**
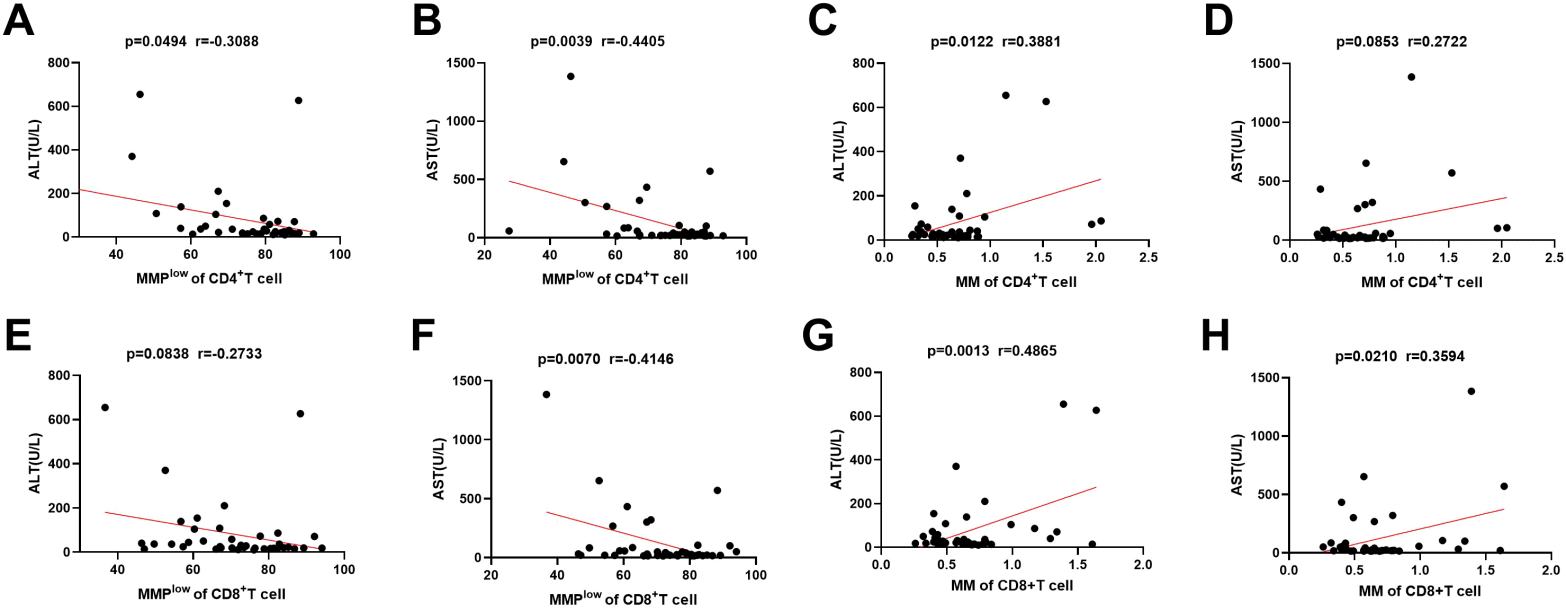
Correlation between MMP^low^ and MM of immune cells and the ALT and AST. **(A–D)** Statistical analysis of the relationship between MMP^low^ and MM of CD4^+^T cell and the ALT and AST. **(E–H)** Statistical analysis of the relationship between MMP^low^ and MM of CD8^+^T cell and the ALT and AST.

In the investigation of mitochondrial function in immune cells and its association with liver injury, the study identified a negative linear correlation between the MMP^low^ ratio of CD4^+^T and CD8^+^T cells in CHB and AST levels (p = 0.0039 and P=0.0070, r = –0.4405 and r = –0.4146; **Figures 3B, F**). And weak negative correlation between the MMP^low^ ratio of CD4^+^T cell and AST levels (p=0.0494, r=-0.3088; **Figure 3A**). However, no correlation was found with MMP^low^ of other lymphocytes (**Figure 3E**; **Supplementary Figures 4A, B**, **4E, F**, **5A, B** and **5E, F**). Conversely, a positive correlation was noted between mitochondrial mass (MM) in CD4^+^T and CD8^+^T cells and ALT levels (p=0.0122 and P=0.0013, r=0.3881 and r=0.4865; **Figures 3C, G**). Additionally, CD8^+^T cell MM showed a positive correlation with AST levels (p=0.0210, r=0.3594; **Figure 3H**), while MM of other lymphocytes did not correlate with ALT or AST levels (**Figure 3D**; **Supplementary Figures 4C, D**, **4G, H**, **5C, D**, and **5G, H**). These findings suggest that variations in mitochondrial membrane potential and mass impact lymphocyte function and are significant in hepatic immune injury in HBV patients.

### 3.5 Association between MMP^low^ and MM of lymphocyte subsets and liver inflammation

**Figure 4:**
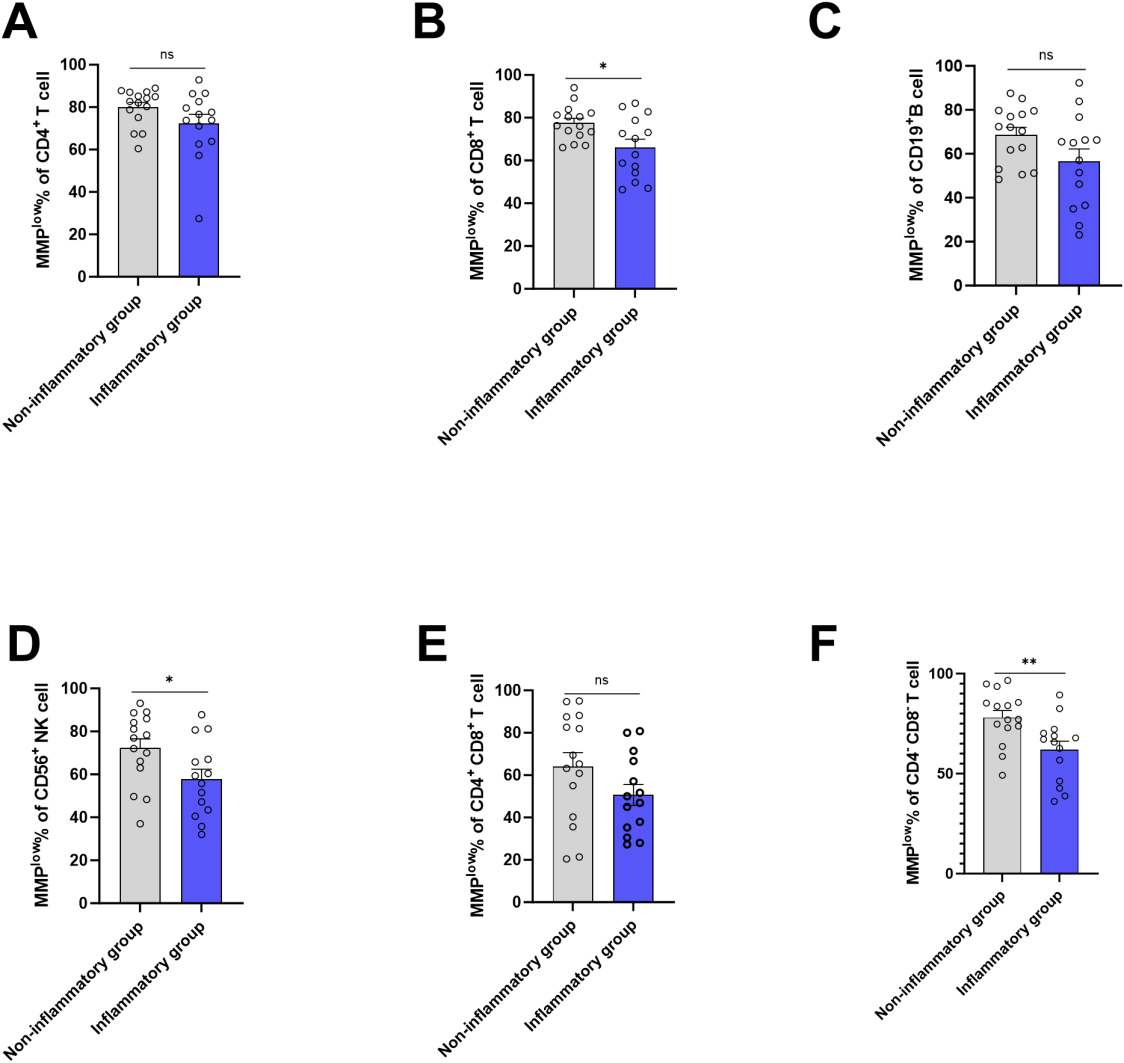
Frequencies of MMP^low^ in the HC group, CHB group and LC group. **(A)** Statistical analysis of the frequencies of MMP^low^ of CD4^+^T cell in inflammatory group and non-inflammatory group; **(B)** Statistical analysis of the frequencies of MMP^low^ of CD8^+^T cell in inflammatory group and non- inflammatory group. **(C)** Statistical analysis of the frequencies of MMP^low^ of CD19^+^B cell in inflammatory group and non- inflammatory group; **(D)** Statistical analysis of the frequencies of MMP^low^ of CD56^+^NK cell in inflammatory group and non- inflammatory group. **(E)** Statistical analysis of the frequencies of MMP^low^ of CD4^+^ CD8^+^T cell in inflammatory group and non- inflammatory group; **(F)** Statistical analysis of the frequencies of MMP^low^ of CD4^-^CD8^-^T cell in inflammatory group and non- inflammatory group. *:p<0.05,**:p<0.01 and ns:p>0.05.

Based on the presence of liver inflammation observed on B-ultrasound, the CHB group (ALT<45) was categorized into inflammation group(n=14) and non-inflammation group(n=15). Using flow cytometry, MMP^low^ of peripheral blood lymphocyte subsets was measured, revealing that the percentage of CD8^+^T cell and CD56^+^ NK cells MMP^low^ in inflammation group (66.13 ± 14.27 and 57.77 ± 17.40) was lower than that in the non-inflammation group (77.59 ± 8.079 and 72.25 ± 16.69), with a statistical difference(p=0.0122 and p=0.0302) (**Figures 4B, D**). The MMP^low^ percentage of CD4^-^CD8^-^T cells in the inflammation group (61.98 ± 15.98) was significantly lower than in the non-inflammation group (78.06 ± 13.42, p=0.0066, **Figure 4F**) the MMP^low^ percentage of CD4+T cells, CD19+B cells and CD4+CD8+T cells had no statistical difference between non-inflammation group and inflammation group (**Figures 4A, C, E**). No differential expression of MM in lymphocyte subsets was identified between the inflammation group and the non-inflammation group (**Supplementary Figure 6**) within the study. Mitochondrial metabolism is an important way for cell function, mitochondria control the ATP production and mitochondrial calcium (Ca2+) uptake by changing MMP and further affect the protein synthesis (18). Tan (19) and Lim (20) found antigen stimulation can change the MMP of DC and CD4^+^T cells, affecting the synthesis of cytokines. Our study found that inflammatory patients had a lower ratio of mitochondrial MMP^low^ in lymphocytes, with high membrane potential that could increase the ATP and ROS production, promote the synthesis and release of inflammatory cytokines, and cause inflammation.

### 3.6 MMP^low^ and MM of lymphocytes in predicting early liver inflammation

**Figure 5:**
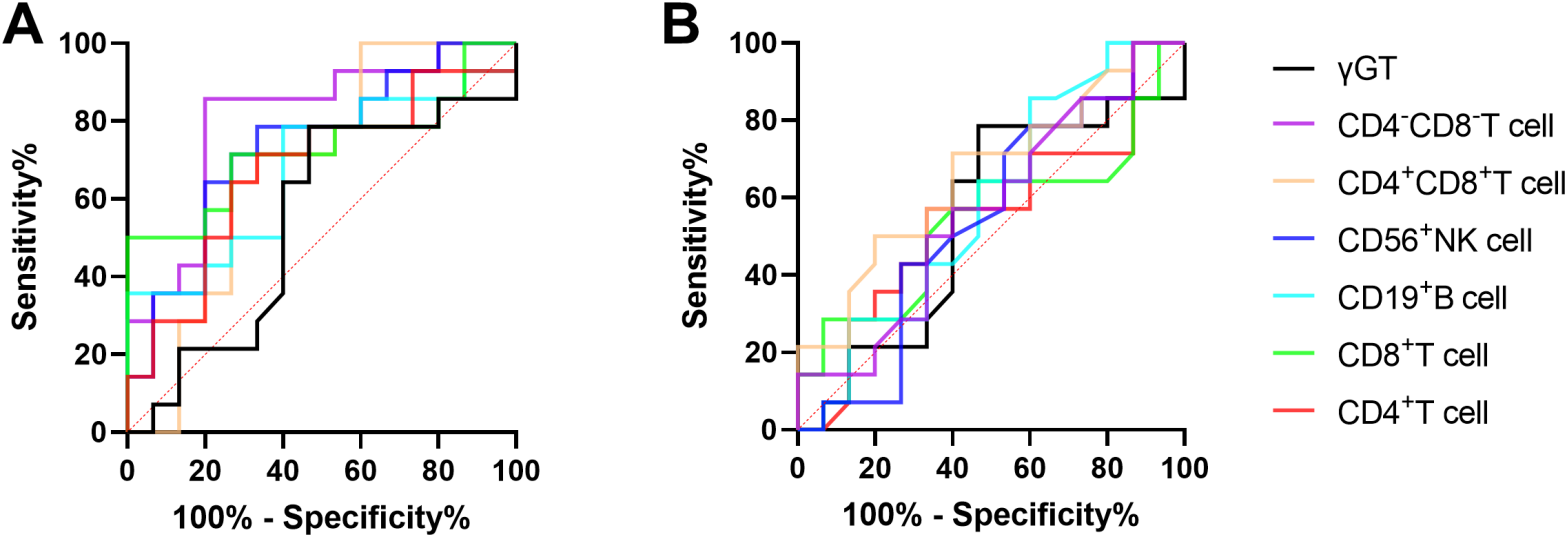
**(A)** The ROC curve of immune cells MMPlow for the difference between inflammatory group and non- inflammatory group. **(B)** The ROC curve of immune cells MM for the difference between inflammatory group and non- inflammatory group.

In the study, ROC curve analysis was conducted to assess the diagnostic efficacy of MMP^low^ and MM of lymphocytes in early liver inflammation, distinguishing between the inflammation group and the non-inflammation group. The AUC of MMP^low^ of CD4^-^CD8^-^T cells, CD56^+^NK cells and CD8^+^T cells were 0.8408, 0.7381 and 0.7286 respectively (p=0.0052, 0.0291 and 0.0362). These indicated a diagnostic value for early liver inflammation, but other indicators have no clinical value. The MMP^low^ of CD4^-^CD8^-^T cells had the highest AUC value and demonstrated the ability to detect liver injury at an earlier stage compared to traditional biomarkers such as ALT and γGT (refer to **Figure 5** and **Table 3**).

**Table 3:** MMP^low^ and MM of lymphocyte subsets distinguished the correlation indexes of ROC curves between hepatic impairment and Non-hepatic impairment in CHB patients(ALT<45U/L).

	AUC	95%*CI*	p value
γGT	0.5452	0.3252 to 0.7653	0.6784
CD4^+^T cell MMP^low^	0.6762	0.4742 to 0.8782	0.1064
**CD8^+^T cell MMP^low^**	**0.7286**	**0.5332 to 9239**	**0.0362**
CD19^+^B cell MMP^low^	0.6762	0.4738 to 0.8786	0.1064
**CD56^+^NK cell MMP^low^**	**0.7381**	**0.5536 to 0.9226**	**0.0291**
CD4^+^CD8^+^T cell MMP^low^	0.6810	0.4787 to 0.8832	0.0972
**CD4^-^CD8^-^T cell MMP^low^**	**0.8408**	**0.6389 to 0.9706**	**0.0052**
CD4^+^T cell MM	0.5500	0.3329 to 0.7671	0.6468
CD8^+^T cell MM	0.5452	0.3252 to 0.7653	0.6784
CD19^+^B cell MM	0.5833	0.3716 to 0.7951	0.4450
CD56^+^NK cell MM	0.5476	0.3316 to 0.7636	0.6625
CD4^+^CD8^+^T cell MM	0.6619	0.4614 to 0.8624	0.1378
CD4^-^CD8^-^T cell MM	0.5690	0.3569 to 0.7812	0.5268

The bold values means statistical difference.

## 4 Discussion

The advancements in research on the mechanism of mitochondrial injury in liver diseases have led to a shift toward intervention strategies targeting mitochondria as a new approach to treating various liver conditions (21). Key determinants of cell death include mitochondrial membrane potential and calcium homeostasis (22). The accumulation of calcium ions not only diminishes ATP production but also induces the excessive opening of the mitochondrial permeability transition pore (MPTP), leading to the collapse of mitochondrial membrane potential (23). Reduced mitochondrial membrane potential significantly contributes to mitochondrial damage, subsequently triggering intracellular ROS generation, signaling apoptosis, and releasing inflammatory factors and mtDNA, culminating in localized liver tissue inflammation (14). This process plays a pivotal pathological role in hepatocyte apoptosis and liver inflammation.

The role of immune-mediated injury in liver damage among patients with HBV infection is significant. Currently, there is limited research on the correlation between mitochondrial damage in immune cells and liver injury in these patients. Studies have revealed that the presence of HBV in immune cells can cause a reduction in the mitochondrial membrane potential of these cells, and hepatitis B antigen has been associated with membrane potential impairment (15). However, the relationship between lymphocyte mitochondrial membrane potential and liver inflammation has not been reported, and there is a lack of research where mitochondrial membrane potential serves as a clinical detection parameter for HBV infection.

In our research, it was observed that the MMP^low^ percentage of CD8^+^T cells exhibited consistent variations with the progression of the disease. The MMP^low^ percentage of CD8^+^T cells was significantly lower in the LC compared to the HC and CHB groups, and the MMP^low^ percentage of CD56^+^NK cells in the LC group was also lower than that in the healthy control group. Our analysis revealed that the mitochondrial mass of CD4^+^CD8^+^T cells in the CHB group was lower than that in the normal healthy control group. These findings indicate that as the disease advances, the membrane potentials of CD8^+^T cells and NK cells that dominate viral immunity are elevated, suggesting an activated state of these cells. Changes in mitochondrial mass and membrane potential are significant contributors to increased reactive oxygen species (ROS) production. Fisicaro et al. (24) identified ROS as a key marker of HBV infection inflammation, increased production of ROS by dysfunctional mitochondria can lead to DNA damage, and superoxide production (MitoSOX) can increase the function of T cells. We also analyzed the relationship between lymphocyte membrane potential and ROS. We found the ROS expression of CD45^+^ MMP^low^ cells was lower than MMP^high^ cells, and the expression of ROS in CD4^+^T cells, CD8^+^T cells and CD56^+^NK cells in patients in the inflammatory group was higher than that in the non-inflammatory group, but there were no statistical difference (**Supplementary Figure 7**). The specimens of **Supplementary Figure 7** were newly collected. Its sample size was smaller than that used in other figures. The relationship between MMP and ROS in CHB needs to be further studied and verified by larger samples. Therefore, further exploring the relationship between membrane potential and ROS or ATP, analyzing the function of MitoSOX and DNA damage in regulating T cells during HBV infection will provide a deeper understanding of the biological significance of mitochondrial function in immune cells and offer additional biomarkers for the clinical diagnosis of CHB and LC.

The relationship between immune cell membrane potential and early liver inflammation was further analyzed in light of the understanding that abnormal immune cell function is a key factor in liver injury. It was observed that changes in immune cell membrane potential may serve as a more sensitive and accurate indicator of liver inflammation than conventional markers such as ALT and AST, which are released after liver injury. The study revealed a linear negative correlation between the MMP^low^ ratio of CD4^+^T and CD8^+^T cells in the CHB group and AST levels. Furthermore, a positive correlation was identified between the MM of CD4^+^T cells and CD8^+^T cells and ALT levels. The MMP^low^ percentage of CD8^-^CD4^-^T cells was found to be decreased in CHB patients with liver inflammation detected via ultrasonography but normal ALT levels.

In the study, it was also observed that CD8^+^T cells, CD56^+^ NK cells, and CD4^-^CD8^-^ T cells in the non-inflammatory group exhibited a higher proportion of low mitochondrial membrane potential. This finding may be attributed to the need for increased mitochondrial production and energy availability during lymphocyte activation. Elevated membrane potential enhances activity within the mitochondrial respiratory electron transport chain, resulting in activated lymphocytes displaying greater mitochondrial membrane potential enrichment. This enrichment supports lymphocyte activation and contributes to a more robust inflammatory response.

This result suggests that the participation of mitochondria may also be required for the polarization of T cells, Similar to the study by Xiao et al. in tumors (23). T cell mitochondrial function may be involved in immune damage by influencing T cell function (17). Different T cell subsets are involved in different stages of liver inflammation, and it is particularly noteworthy that mitochondrial dysfunction of CD3^+^CD4^-^CD8^-^ T cells can occur in early liver inflammation. Activated CD3^+^CD4^-^CD8^-^T cells were divided into cytotoxic and proinflammatory subgroups (25, 26), this study further found that the MMP^low^ percentage of CD3^+^CD4^-^CD8^-^T cells can reflect early liver inflammation. The above results may be related to the involvement of CD3^+^CD4^-^CD8^-^T cells in innate immunity and pro-inflammatory, but there were different subsets of CD3^+^CD4^-^CD8^-^T (25), so further research on the changes of mitochondrial function in different subsets of CD3^+^CD4^-^CD8^-^T cells will further clarify the mechanism of T cells in HBV infection.

The existing studies on mitochondrial dysfunction in HBV found HBV-specific CD8^+^T cells had extensive mitochondrial alterations and increased production of ROS by dysfunctional mitochondria can lead to DNA damage (24, 27), and further studies found replenishment of NAD could restore HBV-specific CD8^+^T cell functions (28). The above and our studies all suggest the HBV infection can cause mitochondrial dysfunction, but our study focuses on factors that cause mitochondrial function changes such as potential and mass, while other studies explore the products of mitochondrial function changes such as ROS and ATP, and we also found NK and CD3^+^CD4^-^CD8^-^T cells had mitochondrial dysfunction which was not explored in other studies.

T cell exhaustion and NK cell function and differentiation are associated with HBV infection. Yan Jia et al. found HBV DNA polymerase upregulated the transcription of PD-L1 and suppressed T cell activity (29). HBV infection mice were used to show that metabolic abnormalities in dysfunctional CD8^+^T cells were the manifestation of prolonged antigenic stimulation (30). In the NK cell, the study also found cell phenotypic alteration and dysfunctional state post hepatitis B subviral particle stimulation in CHB patients. Mitochondria are the main organelles that control cell metabolism, and this study found that the mitochondrial potential and quality of circulating lymphocytes in CHB patients were altered. This study suggests that mitochondrial energy may be related to T cell exhaustion and NK cell function changes, which provides a direction for further revealing the immune tolerance mechanism caused by HBV infection.

The study has several limitations. Firstly, the results require validation with a larger sample size. Secondly, data from HCC patients are absent. Additionally, the relationship between mitochondrial damage and antigen-specific T-cell function during HBV infection needs further investigation, and the study of the relationship between potential and ROS and ATP were necessary to further clarify the role of lymphocyte mitochondria in HBV infection. Consequently, future research should focus on conducting multicenter studies throughout the entire HBV infection cycle, specifically examining mitochondrial aspects of immune cells. Further exploration of mitochondrial mechanisms in HBV infection using animal and cell models is warranted.

## 5 Conclusion

Lymphocyte-induced immune damage is an important mechanism for the occurrence of HBV-infected liver inflammation. The mitochondrial mechanism of lymphocyte activation has been discovered and has been implicated in the development of tumor immunity and autoimmune diseases. Studies have found that HBV infection can cause abnormal mitochondrial metabolism in liver cells, but there is a lack of research on lymphocyte mitochondria and HBV infection, this study conducted a systematic analysis of the percentage of lymphocyte MMP^low^ and the MM in different stages of HBV infection to investigate their correlation with early liver inflammation. In this study, we found that the MMP^low^ percentage of CD8^+^T cells and NK cells changed regularly at different stages of HBV infection. The MMP^low^ and MM of T cells were correlated with liver inflammatory markers such as ALT and AST. This study also aimed to establish the clinical diagnostic value of MMP^low^ and MM in HBV infection and found the MMP^low^ and MM of CD8^+^T cells could be used as a potential indicator to distinguish LC from CHB. The MMP^low^ of CD3^+^CD4^-^CD8^+^T cells had clinical value in reflecting early liver inflammation. This study contributes to a deeper understanding of the role of T cells in liver inflammation, provides clinical data for further research on the mechanism of T cell activation and provides a potential diagnostic indicator for clinical cirrhosis and early liver injury.

## Data Availability

The datasets presented in this study can be found in online repositories. The names of the repository/repositories and accession number(s) can be found in the article/**Supplementary Material.**
