# Supplementary material for: Altered mitochondrial mass and low mitochondrial membrane potential of immune cells in patients with HBV infection and correlation with liver inflammation
Source: Front Immunol. 2024 Nov 22;15:1477646. doi: 10.3389/fimmu.2024.1477646 (PMC11621101; doi:10.3389/fimmu.2024.1477646)
Supplement: Supplementary file 1 [file DataSheet1.pdf]

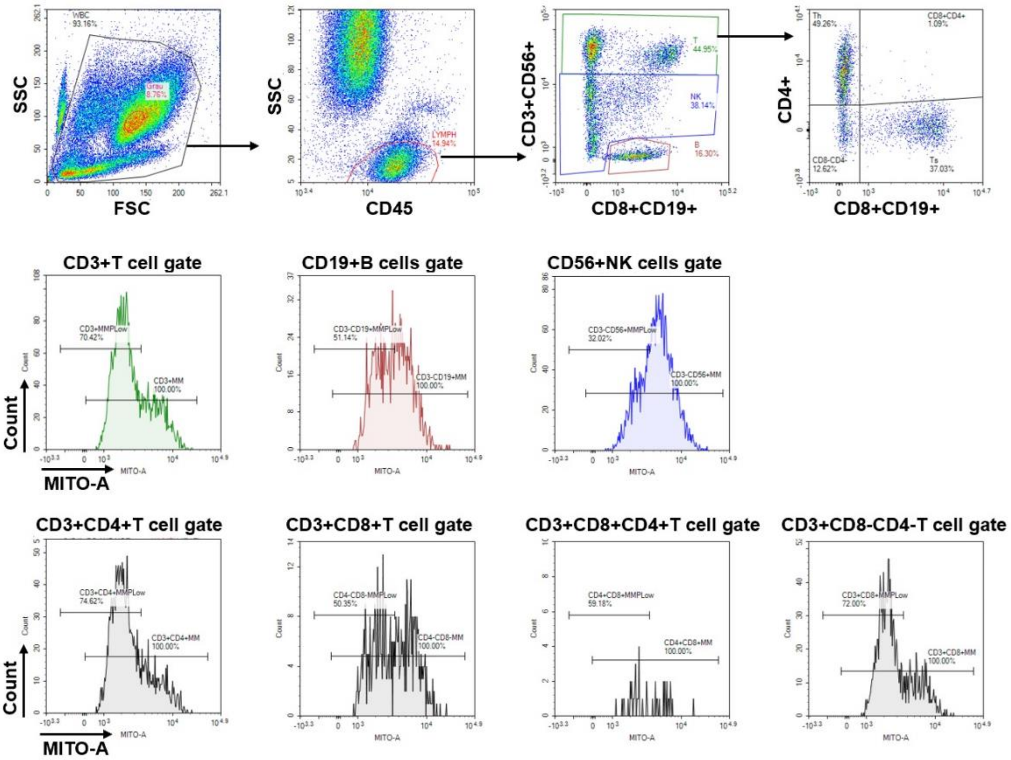

Supplementary material Figure 1: Representative flow cytometric plots showing the gating strategy for Lymphocyte subsets, MM and MMP^{low} expression.

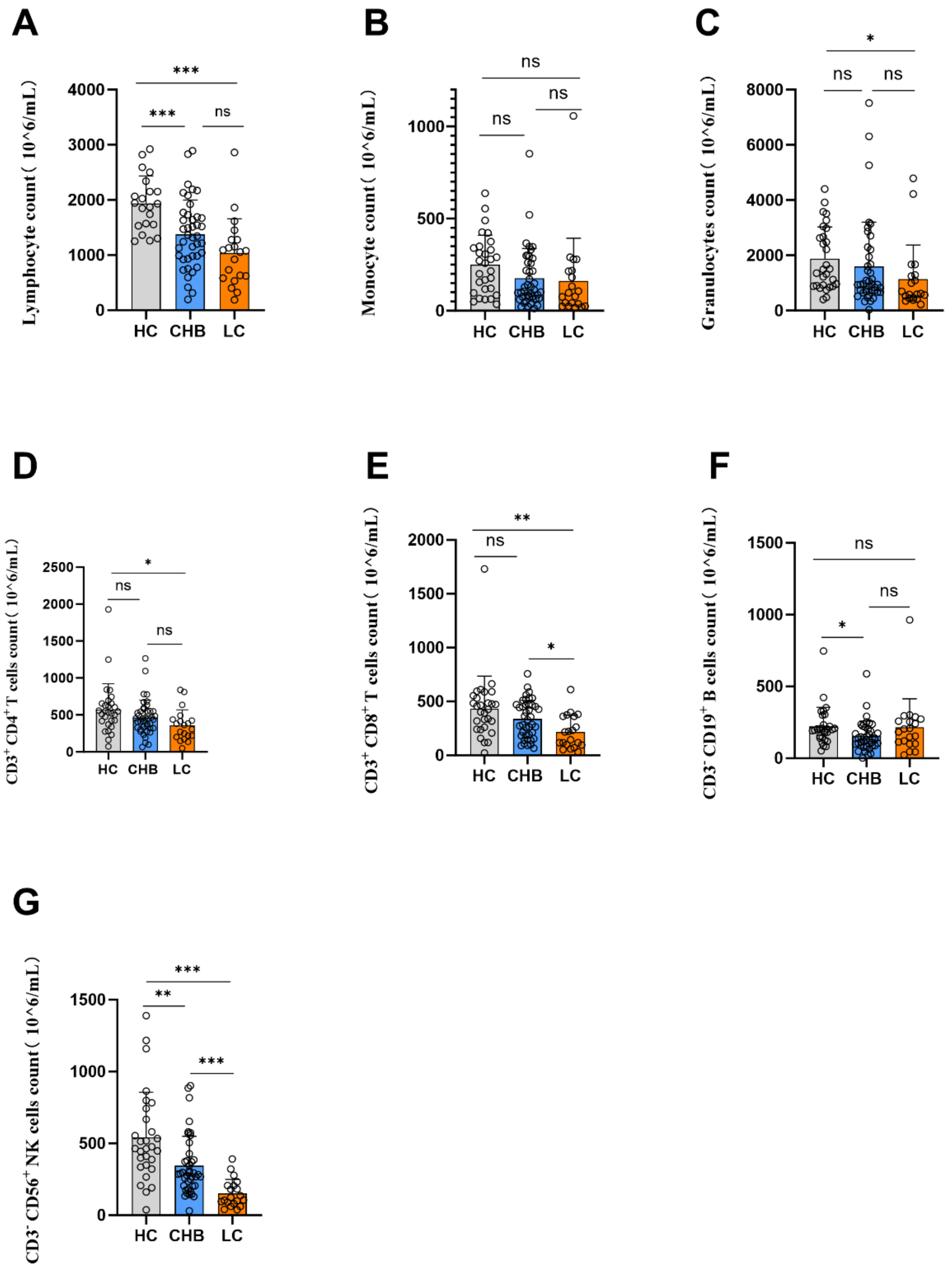

Supplementary material Figure 2: Lymphocyte subset count in the HC group, CHB group and LC group. (A-H) Statistical analysis of the lymphocyte subset count in the HC group, CHB group and LC group. ns:p>0.05, *p<0.05, **<0.01 and ***p<0.001.

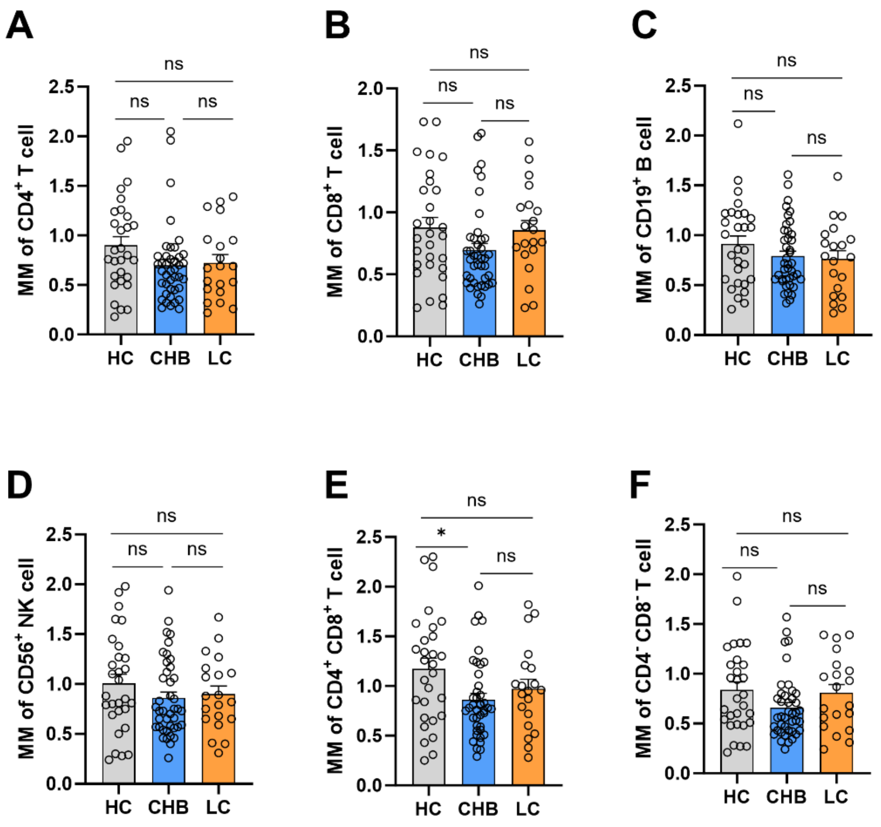

Supplementary material Figure 3: Frequencies of MM in the HC group, CHB group and LC group. (A-F) Statistical analysis of the frequencies of MM in the HC group, CHB group and LC group. *p<0.05, and ns:p>0.05.

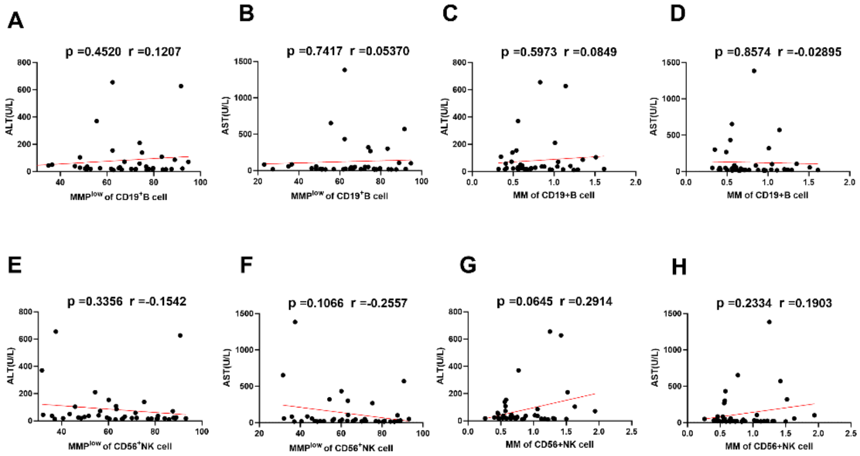

Supplementary material Figure 4: Correlation between MMP^{low} and MM of immune cells and the ALT and AST. (A-D) Statistical analysis of the relationship between MMP^{low} and MM of $CD19^{+}$B cell and the ALT and AST. (E-H) Statistical analysis of the relationship between MMP^{low} and MM of $CD56^{+}$NK cell and the ALT and AST.

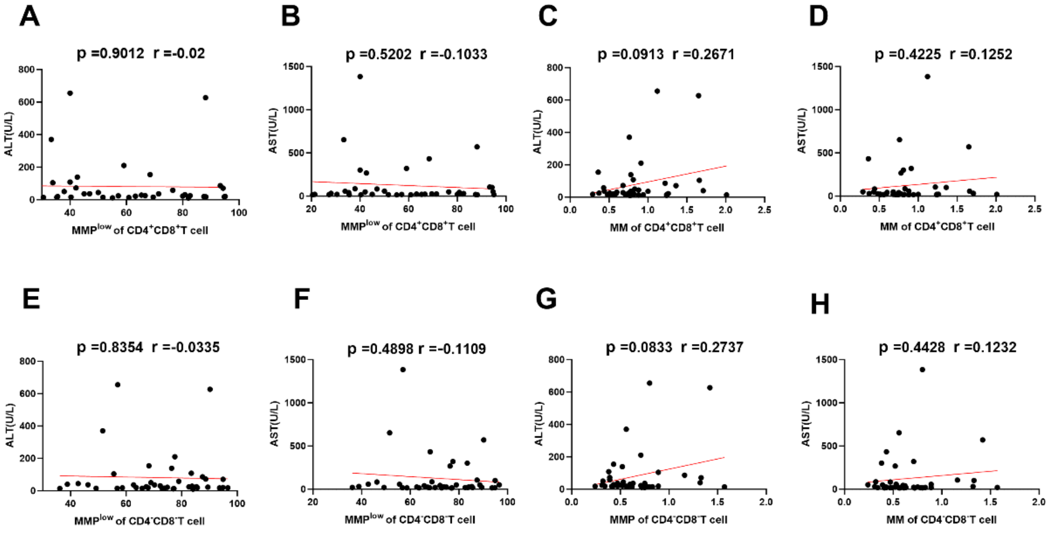

Supplementary material Figure 5: Correlation between MMP^{low} and MM of immune cells and the ALT and AST. (A-D) Statistical analysis of the relationship between MMP^{low} and MM of $CD4^{+}CD8^{+}$T cell and the ALT and AST. (E-H) Statistical analysis of the relationship between MMP^{low} and MM of $CD4^{-}CD8^{-}$T cell and the ALT and AST.

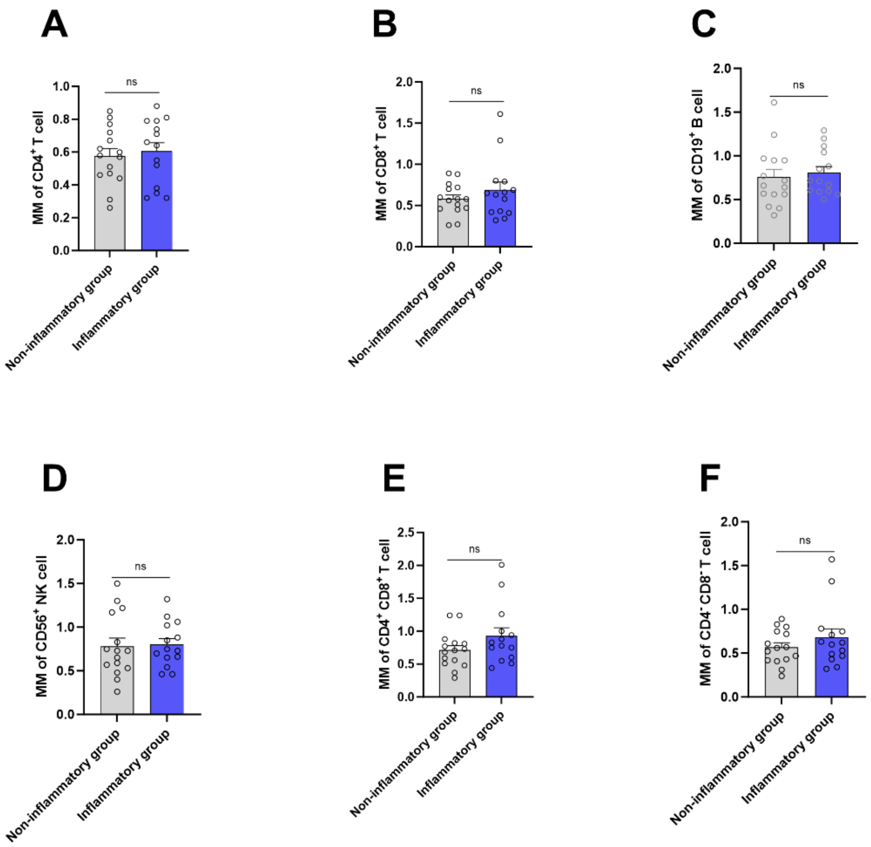

Supplementary material Figure 6: Frequencies of MM in the inflammation group and non-inflammation group. (A-F) Statistical analysis of the frequencies of MM in the inflammatory group and non- inflammatory group. ns:p>0.05.

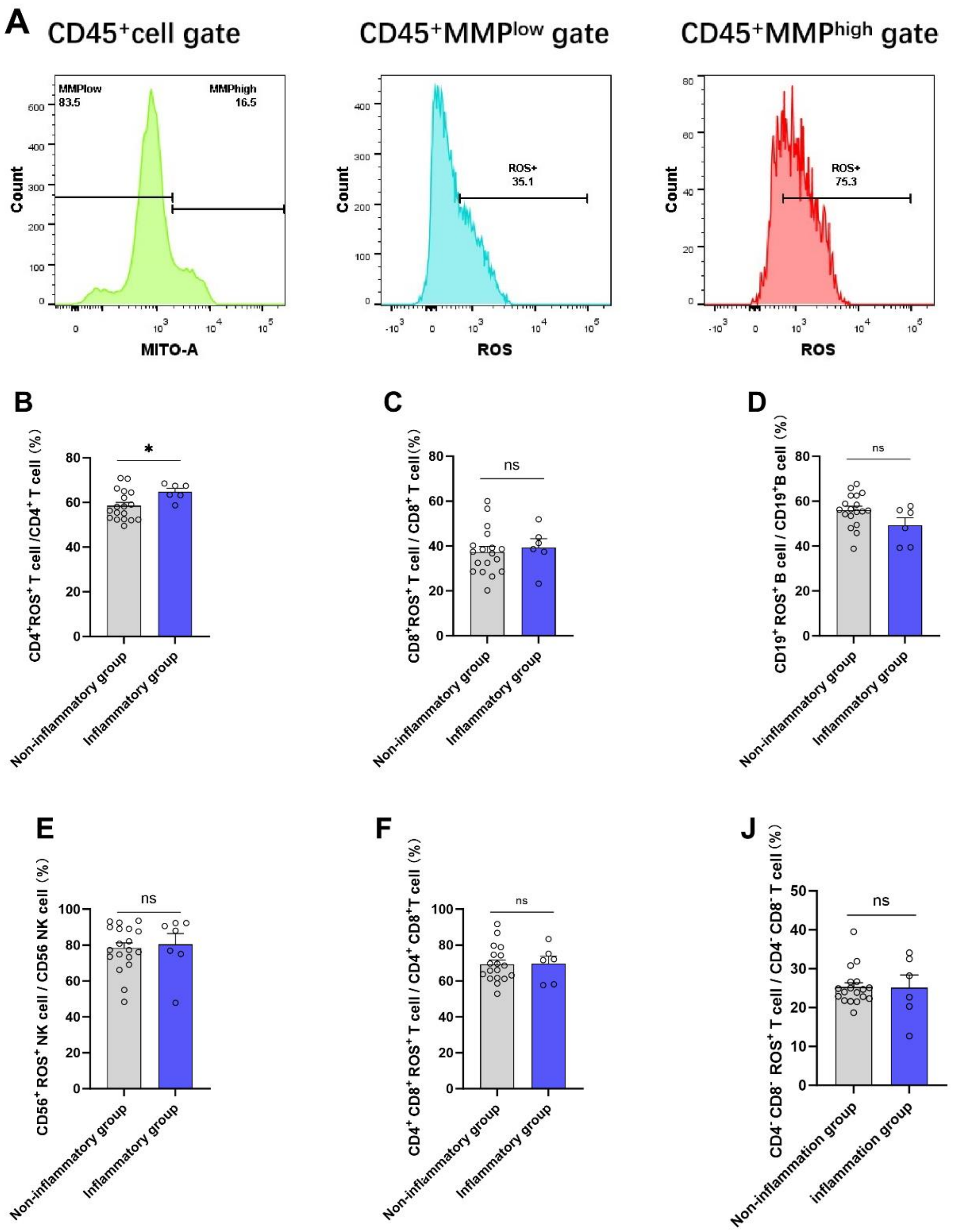

Supplementary material Figure 7: Frequencies of ROS in the inflammation group and non-inflammation group. (A) Representative flow cytometric plots showing the gating strategy for ROS, MMP^{high} and MMP^{low} expression. (B-J) Statistical analysis of the frequencies of ROS in the inflammatory group and non- inflammatory group. *$p<0.05$ and ns:$p>0.05$.
